# A Neonatal Salt-Wasting Crisis Mimicking Congenital Adrenal Hyperplasia: A Case of Transient Pseudohypoaldosteronism

**DOI:** 10.7759/cureus.107777

**Published:** 2026-04-27

**Authors:** Tuqa A Abdulsalam, Tasneem A Obaisi

**Affiliations:** 1 Pediatric Endocrinology, Al Jalila Children's Specialty Hospital, Dubai, ARE; 2 Pediatrics, Jordanian Royal Medical Services, Amman, JOR

**Keywords:** acute kidney injury, congenital adrenal hyperplasia, hydronephrosis, neonatal salt-wasting crisis, obstructive uropathy, pseudohypoaldosteronism, secondary pseudohypoaldosteronism, transient pseudohypoaldosteronism, urinary tract anomaly, vesicoureteral reflux

## Abstract

Salt-losing crisis in the neonate is a life-threatening event manifested by hyponatremia, hyperkalemia, and metabolic acidosis. Whilst this biochemical finding is classically seen in congenital adrenal hyperplasia (CAH), conditions of aldosterone action, such as pseudohypoaldosteronism (PHA), can also present with these findings but require different care. A four-week-old term male infant is described who was brought in with vomiting, lethargy, and anuria. At the time of admission, he was in shock with marked hyponatremia (119 mmol/L), life-threatening hyperkalemia (9.8 mmol/L), metabolic acidosis, and acute kidney injury (AKI). He needed resuscitation, treatment of hyperkalemia, and admission to pediatric intensive care. CAH was considered at the beginning, but newborn mass screening for 17-hydroxyprogesterone was normal, and serum cortisol before steroid therapy was adequate, so primary adrenal failure may be ruled out. Electrolytes and renal function returned promptly to normal after volume repletion, and the patient passed into a polyuric recovery. The clinical course, including rapid resolution of PHA, was consistent with transient (secondary) PHA related to severe volume depletion and prerenal AKI, resulting in temporary renal tubular resistance to aldosterone. This case illustrates the necessity to differentiate secondary PHA from CAH in neonates presenting with salt loss crises, since early recognition spares lifelong unnecessary steroid exposure and directs appropriate supportive care.

## Introduction

Neonatal salt-losing crises are emergencies that must be quickly diagnosed, while severe hyponatremia and hyperkalemia can precipitate seizures, arrhythmias, hypovolaemic shock, and death. The quintessential biochemical profile of hyponatremia, hyperkalemia, and metabolic acidosis in early infancy is predominantly caused by salt-wasting congenital adrenal hyperplasia (CAH), predominantly 21-hydroxylase deficiency. Accordingly, CAH is the first diagnostic suspicion associated with this electrolyte profile. However, this pattern is caused by a dysfunction of mineralocorticoid action, rather than by a single disease process, and different disorders of aldosterone synthesis or action may present with the same pattern [1].

Aldosterone is critical for sodium retention and potassium loss in the distal nephron via mineralocorticoid receptor-epithelial sodium channels (ENaC) activation. Inactivation of this pathway leads to salt loss in the kidney. In contrast to CAH, in which aldosterone synthesis is decreased because of defects in the steroidogenesis pathway involving insufficient enzymatic activity, pseudohypoaldosteronism (PHA) refers to renal tubular unresponsiveness to aldosterone despite normal or high aldosterone levels. The presence of these mechanisms can be clinically significant, since CAH often necessitates lifelong glucocorticoid and mineralocorticoid supplementation, while PHA is predominantly treated with sodium supplementation, and electrolyte imbalances are corrected without chronic use of steroids [2,3].

PHA can be either primary (genetic) or secondary (acquired). Mutations targeting the mineralocorticoid receptor or ENaC subunits give rise to primary forms that usually manifest in early infancy with recurrent episodes of salt wasting [3]. In contrast, secondary or transient PHA is a condition with a reversible cause, such as a urinary tract infection, pelvic obstructive uropathy, renal inflammation, or extreme dehydration. In these circumstances, renal tubular derangement results in transient aldosterone resistance and salt wasting. Of note, mineralocorticoid responsiveness generally recovers when renal perfusion and tubular function normalize [2-4].

Since neonatal salt-wasting refers to a heterogeneous group of disorders with common biochemical profiles, diagnosis based solely on electrolyte patterns would be misleading. Newborn screening for CAH has facilitated early diagnosis but does not exclude alternative causes of mineralocorticoid impairment despite a normal screen. Recognition of the transitory nature of PHA is, thus, important to prevent prolonged treatment with steroids and to provide supportive care. Herein, we report a case of a baby boy who initially presented with a very severe salt-losing crisis and was thought to be suffering from CAH that turned out to be consistent with transient PHA.

## Case presentation

A male neonate was brought to the emergency department at four weeks of age with feeding refusal after two days of vomiting all non-bilious feeds, with progressively decreased oral intake. The parents stated that, in the 24 hours before hospitalization, he had not passed any urine and was very drowsy. There was no fever, diarrhea, shortness of breath, or sick contacts. The neonate was born via normal vaginal delivery at 39 weeks of gestation, with an uncomplicated antenatal history, a birth weight of 3 kg, and no admission to neonatal intensive care. The newborn screening test, including the 17-hydroxyprogesterone test, was negative. He was fed formula from birth. There was no family history of endocrine or renal disease, although parental consanguinity was later confirmed.

The baby was hypotonic and looked ill, poorly perfused at presentation. Cold peripheries with mottling of the extremities, poor femoral pulse, and slow capillary refill were found on physical examination. Cardiac exam was unremarkable, with no murmurs and normal heart sounds. Lung fields were clear, and abdominal examination was negative for distension and soft to palpation. Impaired peripheral perfusion precluded attainment of peripheral venous access, with a normal male genital without hyperpigmentation.

First record point-of-care from capillary blood gas was indicative of severe metabolic acidosis (pH 7.18, bicarbonate of 13 mmol/L, and base excess -13.9). Serum electrolytes revealed marked hyponatremia (Na of 119 mmol/L) and life-threatening hyperkalemia (K of 9.8 mmol/L). Serum glucose was 82 mg/dL. The renal profile demonstrated elevated serum creatinine (3.16 mg/dL) and urea (134 mg/dL), indicative of acute kidney injury (AKI). Lactate was 4.7 mmol/L. The inflammatory parameters were slightly elevated (C-reactive protein (CRP) of 10 mg/L, procalcitonin of 0.25 ng/mL). The child was resuscitated with a total of two 20 mL/kg boluses of 0.9% saline, after which perfusion and heart rate improved. Cardiovascular stability for hyperkalemia was maintained with intravenous calcium gluconate, nebulized salbutamol, sodium bicarbonate (1 mEq/kg), and insulin with dextrose infusions. Presteroid, cortisol, and adrenocorticotropic hormone (ACTH) levels were sampled (Table 1).

**Table 1:** Summary of laboratory findings at presentation and during hospitalization

Parameter	Initial value	Follow-up value in 48 hours	Reference range
pH	7.18	7.40	7.35-7.45
HCO₃⁻ (mmol/L)	13	22	21-28
Sodium (mmol/L)	119	141	134-143
Potassium (mmol/L)	9.8	3.8-5.1	3.4-5.0
Chloride (mmol/L)	89	111	97-108
Lactate (mmol/L)	4.7	1.0	0.5-1.6
Creatinine (mg/dL)	3.16	0.35	0.2-0.4
Urea (mg/dL)	134	23	12-40
Glucose (mg/dL)	82	120	60-100
Calcium (mg/dL)	8.7	9.5	8.6-11
Albumin (g/dL)	3.7	3.3	3.1-5.0
CRP (mg/L)	10	-	<1.3
Procalcitonin (ng/mL)	0.25	-	<0.05
WBC (×10³/µL)	15.9	-	5-19
Hemoglobin (g/dL)	11.5	-	11.5-16.5
Platelets (×10³/µL)	561	-	210-500
Cortisol (nmol/L)	806	-	68-327
17-OH Progesterone (nmol/ L)	17	-	0.39-24.21
Aldosterone (ng/dL)	More than 1000	-	5-90
Renin (uIU/mL)	9.04	-	2.8-39.9

**Figure 1:**
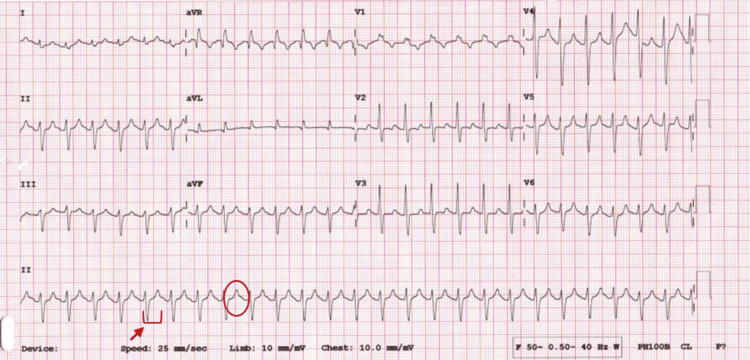
Electrocardiogram demonstrating features consistent with severe hyperkalemia, including widened QRS complexes (circle) and prolonged QT interval (arrow)

The patient was empirically started on meropenem for presumed sepsis, along with an IV stress dose of hydrocortisone 25 mg once within one hour of presentation. The ECG showed broad QRS complexes; a prolonged QTc interval of 533 milliseconds (msec) was seen in severe hyperkalemia (Figure 1).

Due to the concomitant hyponatremia, hyperkalemia, and metabolic acidosis, we suspected CAH and gave a bolus of hydrocortisone. However, a baseline serum cortisol level before steroid administration (806 nmol/L) argued against primary adrenal insufficiency. Hence, attention was shifted to pseudohypoaldosteronism.

**Figure 2:**
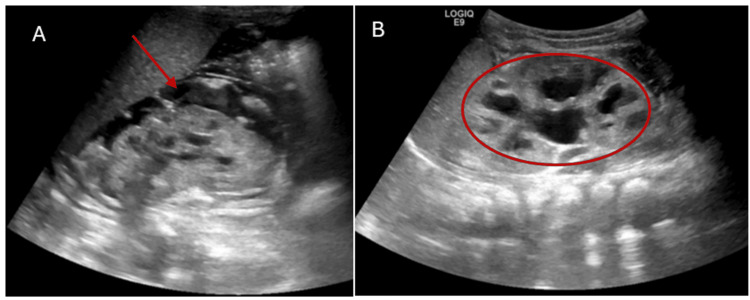
Bilateral hydroureteronephrosis (circle) with perinephric collection (arrow) (A) Right kidney and (B) left kidney

Renal profile and acid-base status had largely corrected within 24-36 hours following hydration and electrolyte replacement. Serum creatinine decreased by 1.2 mg/dL and stabilized at 0.35 mg/dL. Hyperkalemia resolved, and the sodium level gradually normalized. Upon admission to the pediatric intensive care unit, a Foley catheter was placed for strict control of urine output, which showed profound polyuria with urine output up to 20 mL/kg/hour. This raised suspicion of underlying renal pathology. Renal ultrasound showed bilateral hydronephrosis, and a urinary tract anomaly was suspected to be another cause of the electrolyte imbalance (Figure 2).

**Figure 3:**
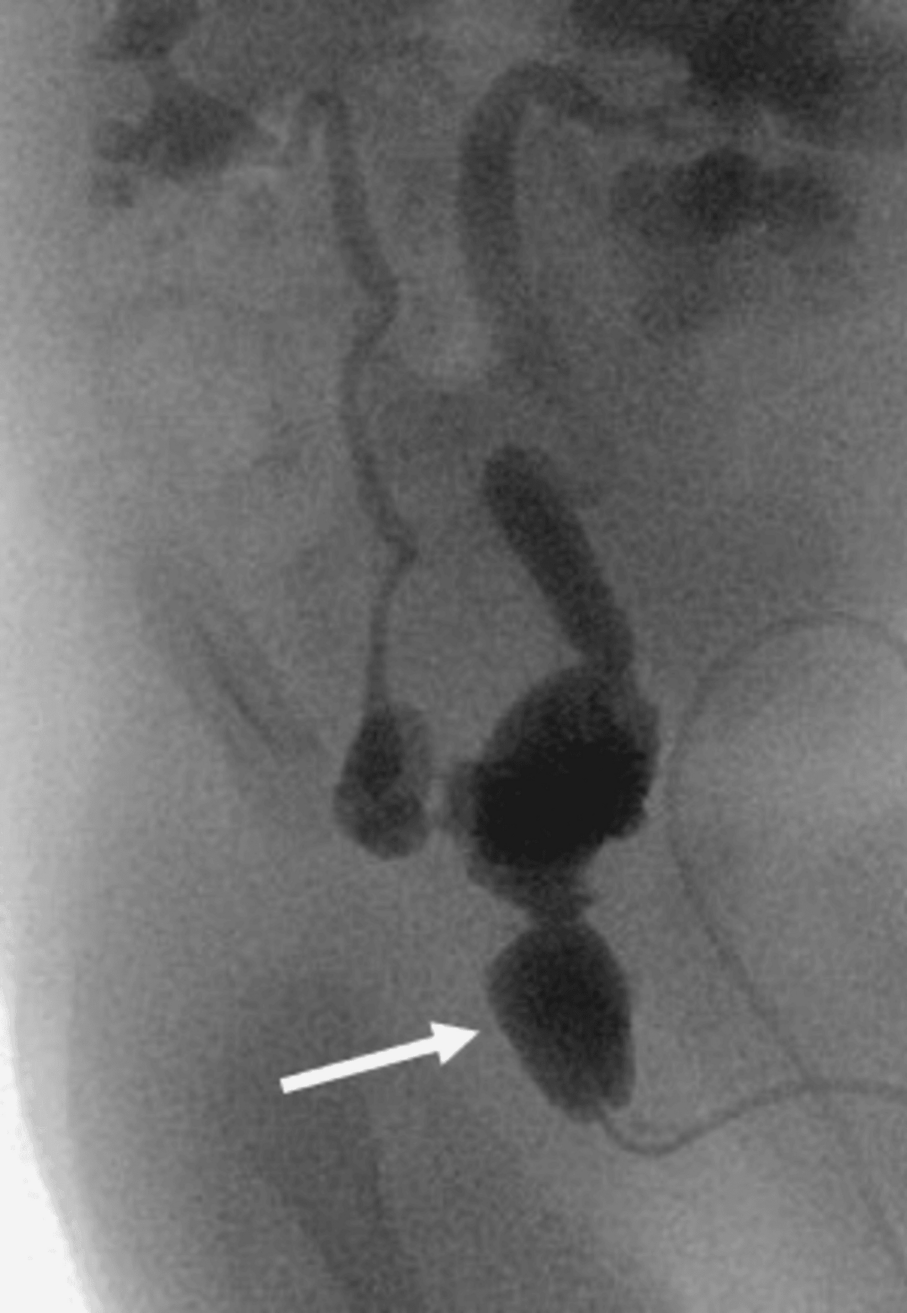
Findings are consistent with bladder outflow obstruction due to severe posterior urethral valve, causing significantly reduced bladder capacity, bilateral grade 5 reflux, and characteristic posterior urethral dilation (arrow)

A voiding cystourethrogram (VCUG), performed and captured once the patient was clinically stable and electrolyte abnormalities corrected, showed vesicoureteral reflux (VUR) (Figure 3).

The diagnosis of VUR returned another clue to the secondary PHA caused by urinary tract anomaly and the dysfunction of the renal tubule. Given the substantial reflux and potential for further renal damage, the patient was referred for surgical care with eventual corrective intervention.

The clinical picture is most compatible with transient (secondary) PHA that was probably precipitated by severe dehydration and prerenal AKI, leading to reversible renal tubular resistance to aldosterone. The baby's condition remained clinically stable, with electrolytes normalized at follow-up.

## Discussion

Neonatal salt-wasting crisis is an urgent endocrine emergency with a clinical triad of hyponatremia, hyperkalemia, and metabolic acidosis occurring primarily due to salt-wasting CAH. However, this biochemical picture is neither disease-specific nor produced purely by diseases affecting aldosterone action, as in PHA. It is important to differentiate between these entities, as management strategies and long-term implications differ widely.

As in this case, the initial presentation can closely resemble CAH, and empiric steroid treatment is initiated. A normal newborn screen for 17-hydroxypregesterone and an adequate pretreatment cortisol level effectively ruled out primary adrenal insufficiency. This is consistent with evidence that early detection of CAH has not reduced the need for a differential diagnosis in infants presenting with salt-wasting crises as newborn screening programs become universal [1,2].

PHA consists of renal tubular resistance to aldosterone with normal or elevated hormone levels. It can be classified as primary (or genetic) and secondary (transient). Mutations that affect either the mineralocorticoid receptor or the ENaC cause primary PHA type 1, frequently resulting in persistent and recurrent salt-wasting episodes [3]. In comparison, secondary PHA is most commonly seen in conditions characterized by reversible renal states, which can include obstructive uropathy, urinary tract infections, or severe dehydration that causes distal tubular resistance to aldosterone actions [4,5].

The rapid normalization of electrolytes after fluid resuscitation in our patient strongly favors a diagnosis of transient PHA. Such a clinical course is in keeping with previous reports that restoration of renal perfusion reverses tubular resistance to aldosterone [4]. This case also features VUR, providing potential explanatory underpinnings. Urinary tract anomalies and infections are also well-known causes of secondary PHA, probably because inflammation affects tubular function and sodium reabsorption [5,6].

Hormonal profile is a very important differentiating factor between CAH and PHA. CAH presents with cortisol deficiency and high 17-hydroxyprogesterone, while PHA is characterized by an increase in renin and aldosterone owing to end-organ resistance. The clinical and biochemical improvement with supportive therapy is suggestive of secondary PHA.

This case reveals several important clinical implications. First, a diagnosis based entirely on abnormalities of electrolytes may be incorrect and expose patients needlessly to glucocorticoids and mineralocorticoids. Second, the early recognition of secondary causes (e.g., urinary tract anomalies) is vital to avoid recurrence and prevent long-term renal sequelae. Finally, transient PHA should be entertained in any neonate who presents with salt-wasting, especially if there is an early turnaround with hydration and no biochemistry to suggest adrenal insufficiency.

## Conclusions

This case illustrates that, while neonatal salt-wasting crises are traditionally considered to be due to CAH, they can also occur in reversible conditions, such as secondary PHA. Thorough evaluation, including hormonal studies and therapeutic response, is necessary to prevent misdiagnosis and needless prolonged steroid therapy. The rapid correction of electrolyte derangements with fluid resuscitation combined with identification of an underlying urinary tract anomaly indicates that, in this case, it was transient PHA that led to diagnosis. Close clinical observation and optimized management may be necessary to prevent serious complications and ensure good outcomes in affected neonates.
